# Elevated lactate dehydrogenase predicts poor prognosis of acute ischemic stroke

**DOI:** 10.1371/journal.pone.0275651

**Published:** 2022-10-07

**Authors:** Xia-Xia Jin, Mei-Dan Fang, Ling-Ling Hu, Yuan Yuan, Jiu-Fei Xu, Guo-Guang Lu, Tao Li

**Affiliations:** 1 Department of Clinical Laboratory, Taizhou Hospital of Zhejiang Province, Taizhou Enze Medical Center (Group), Linhai, Zhejiang Province, China; 2 Department of Cardiovascular Medicine, Taizhou Hospital of Zhejiang Province, Taizhou Enze Medical Center (Group), Linhai, Zhejiang Province, China; Chinese Academy of Medical Sciences and Peking Union Medical College, CHINA

## Abstract

**Background:**

Lactate dehydrogenase (LDH) is associated with the prognosis of many diseases, but the relationship between LDH and the poor prognosis (recurrence and death) of acute ischemic stroke (AIS) has not been fully clarified. This study aimed to investigate the association between admission LDH level and poor prognosis in patients with AIS.

**Methods:**

This retrospective study enrolled AIS patients treated in Taizhou Hospital of Zhejiang Province from July 2019 to December 2019. Poor prognosis included AIS recurrence and all-cause death at 3, 6, and 18 months. The correction between LDH and poor prognosis or all-cause death was assessed. Lasso Cox expression and multivariate Cox expression analyses were used to evaluate the association of LDH with the risk of poor prognosis and all-cause death, respectively. A nomogram was constructed to evaluate the predictive Values of LDH for the poor prognosis and all-cause death of AIS.

**Results:**

732 patients were included in the study. Multivariate analysis shows that admission LDH levels were significantly correlated with poor prognosis [odds ratio (OR),1.003; 95% confidence interval (95% CI), 1.001–1.005; P = 0.001] and all-cause death (OR, 1.005; 95% CI, 1.000–1.009; P = 0.031). The correlation analysis showed that admission LDH level was positively correlated with National Institutes of Health Stroke Scale (NIHSS) score and modified Rankin Scale (mRS) score. Time-dependent receiver operating characteristic (td-ROC) curves analysis showed that the AUC values of admission LDH level for predicting prognosis of AIS patients in 3-month, 6-month, 12-month and 18-month were 0.706 (95% CI, 0.604–0.810), 0.653 (95% CI, 0.583–0.723), 0.616 (95% CI, 0.556–60676) and 0.610 (95% CI, 0.552–0.680), respectively. And td-ROC also showed that the AUC values of admission LDH level for predicting all-cause death of AIS patients in 3-month, 6-month,12-month and 18-month were 0.861 (95% CI, 0.764–0.958), 0.824 (95% CI, 0.753–0.890), 0.726 (95% CI, 0.633–0.819) and 0.715 (95% CI, 0.622–0.807), respectively. The nomograms were constructed to create the predictive models of the poor prognosis and all-cause death of AIS.

**Conclusion:**

Higher LDH levels are independently associated with poor prognosis and all-cause death of AIS.

## Introduction

Stroke is the second leading cause of death after heart disease worldwide, and Acute ischemic stroke (AIS) accounts for about 70% of strokes [1,2]. AIS is characterized by a high recurrence rate, high mortality rate, and high disability rate. Some studies have shown that the cumulative all-cause mortality rate 30 days after stroke is 10.5%, 21.2% in 1 year, and 39.8% in 5 years, respectively [3]. A Meta-analysis also reported that the recurrence rate of stroke is 7.7% at 3 months, 9.5% at 6 months, and 10.4% at 1 year, 16.1% at 2 years, respectively, and recurrent cerebral infarction had higher mortality and disability rates [4]. Therefore, it is important to make a preliminary assessment of the patient’s prognosis.

LDH is a glycolytic enzyme widely distributed in human tissues. It is the main participant in glucose metabolism and is released from cells when the cell membrane is damaged [5]. At the same time, LDH is also widely concerned as a biomarker that plays an important role in monitoring the prognosis of diseases. It is reported that high levels of serum lactate dehydrogenase are adverse prognostic factors for many malignant tumors, including urothelial carcinoma, metastatic prostate cancer, metastatic renal cell carcinoma, and lung cancer [6–9]. It is also reported that LDH plays a certain role in predicting the adverse outcomes of preeclampsia and eclampsia and the death of covid-19 patients [10,11].

Studies on the relationship between LDH and cerebrovascular diseases mainly focus on brain injury and intracerebral hemorrhage. Some scholars point out that LDH is a reliable predictor of early hematoma expansion and adverse prognosis in ICH patients [12]. It is also reported that LDH level in cerebrospinal fluid is biomarkers of early brain injury and delayed cerebral ischemia of subarachnoid hemorrhage [13]. Then, there are few reports on the relationship between LDH and AIS, therefore, the purpose of this study was to explore the role of LDH in predicting the adverse prognosis (all-cause death and recurrence) of AIS patients.

## Materials and methods

### Study design

This retrospective and observational study were approved by the Ethics Committee of Taizhou Hospital of Zhejiang Province, China (reference number K20211229). Informed consent was abandoned because it was a retrospective study. The patients with acute ischemic stroke (AIS) hospitalized admitted to Taizhou Hospital of Zhejiang Province from July 2019 to December 2019 were included in this study. The diagnosis of acute ischemic stroke was determined by World Health Organization criteria with confirmation by brain computed tomography (CT) or magnetic resonance imaging (MRI). The inclusion criteria were age ≥18 years and stroke symptom onset within 72 h. The exclusion criteria were as follows: 1) Patients with other diseases, including malignant tumor, hematological disease, several renal or hepatic dysfunction and autoimmune disease, etc; 2) those who had a history of surgery or infection with 90 days; 3) incomplete baseline clinical data; 4) loss to follow-up.

### Data collection

Clinical and laboratory data were obtained from the inpatient medical records and outpatient medical records, including 1) demographics and clinical history including age, sex, Time from onset to admission, drinking, smoking, length of stay, hospitalization costs; 2) medical history including stroke history, Hypertension, Diabetes mellitus, Atrial Fibrillation (AF), Heart failure (HF), Coronary heart disease (CAD); 3) Carotid artery stenosis; 4) stroke etiology according to TOAST classification; 5) Medication in hospital including Intravenous thrombolysis; 6) NIHSS, mRS scores and SPI-Ⅱscore were scored by a neurologist according to the National Institutes of Health Stroke Scale (NIHSS), Modified Rankin Scale (mRS) and Strioke Prognostic Instrument Ⅱ(SPI-Ⅱ); 7) laboratory data including Alanine aminotransferase (ALT), Aspartate aminotransferase (AST), total protein (TP), Albumin (ALB), Fasting blood glucose (FBG), Total cholesterol (TC), triglyceride (TG), Low density lipoprotein (LDL), high density lipoprotein (HDL), creatine kinase (CK), Lactate dehydrogenase (LDH), Hypersensitive C-reactive protein (hs-CRP), D dimer (DD), Fibrinogen (Fib), international normalized ratio (INR), white blood cell count (WBC), Neutrophil count (Neutrophil), Lymphocyte count (Lymphocyte), Monocyte count (Monocyte), red blood cell count (RBC), hemoglobin (Hgb), Red blood cell volume distribution width (RDW), platelet count (PLT), erythrocyte sedimentation rate (ESR) from fasting bloods drawn within 24 hours of admission. And we had access to information that could identify individual participants during or after data collection.

### Study outcomes

Stroke outcome was collected by outpatient review or telephone interview at 3-month, 6-month and 12-month, by phone interview at the 18-month. The poor prognosis in our study includes all-cause death and recurrent stroke. Recurrent stroke was defined as follows, 1) a new neurological impairment; 2) the initial symptoms and signs were aggravated and the time from the first stroke was more than one month; 3) new stroke confirmed by craniocerebral MRI or CT; 4) at least one previous history of stroke.

### Statistical analyses

The categorical data were expressed using counts (percentage), and the numerical variables were expressed using median with interquartile range(IQR). Univariate COX regression analyses were performed to assess the intergroup difference. Time-dependent receiver operating characteristic (td-ROC) curves analysis was performed to determine the area under the curve (AUC) and 95% CI of LDH. Survival curves were drawn using the Kaplan-Meier method, and differences in survival between groups were compared using the log-rank test. Spearman’s correlation analysis was used to assess the correlations between LDH and other indicators. Lasso Cox regression and multivariate COX regression analyses were conducted to evaluate associations between LDH with mortality risk and prognosis after adjustment for possible confounders, involving all variates with a P < 0.05 in initial univariate analysis, with results exhibited as hazard ratio (HR) and 95% confidence interval (CI). The nomograms were created based on the results of multivariate Cox regression analysis. P<0.05 was considered to be statistically significant. All data were statistically analyzed using R (Version: 4.0.5).

## Results

### Patient baseline characteristics

**Fig 1:**
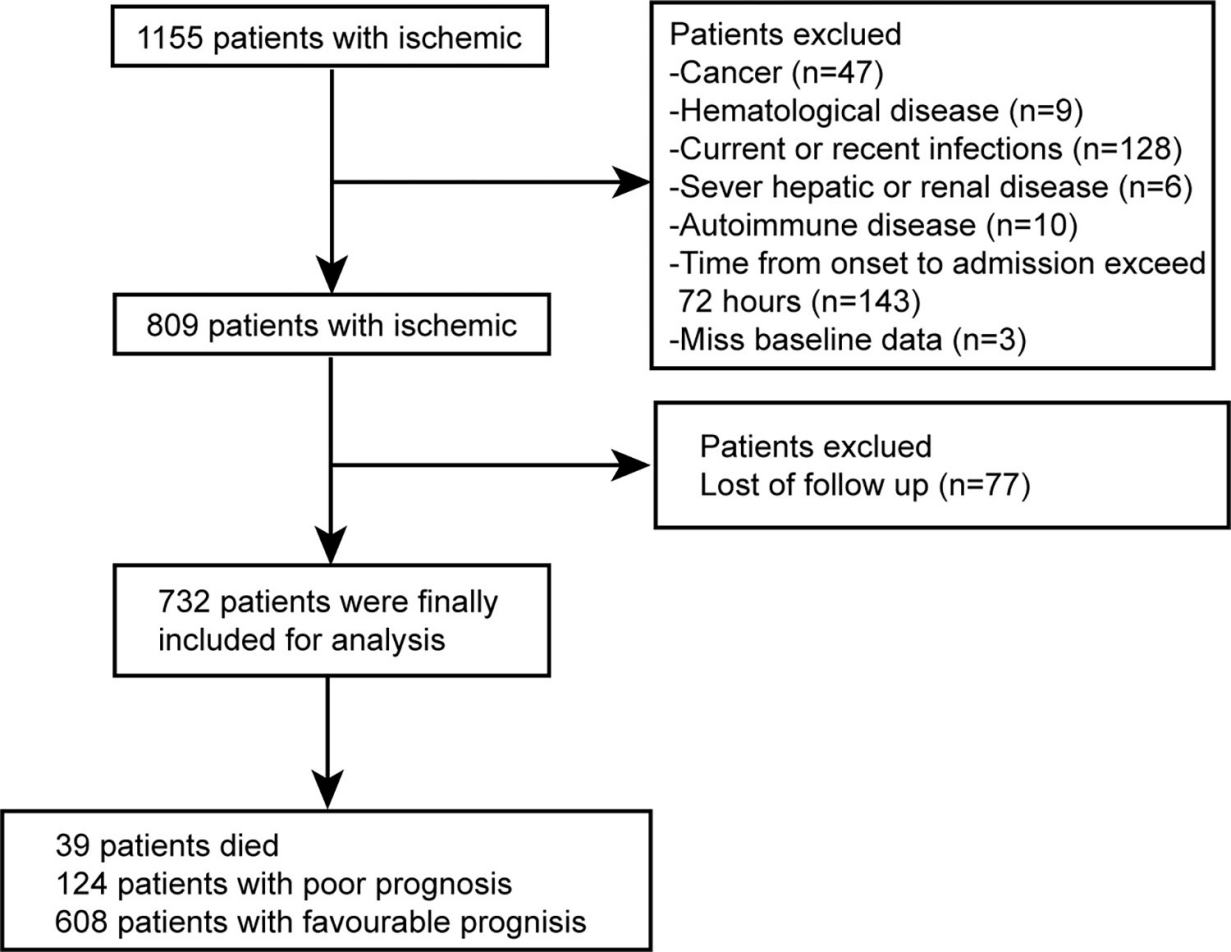
Flow chart of patient enrollment in this study.

**Fig 2:**
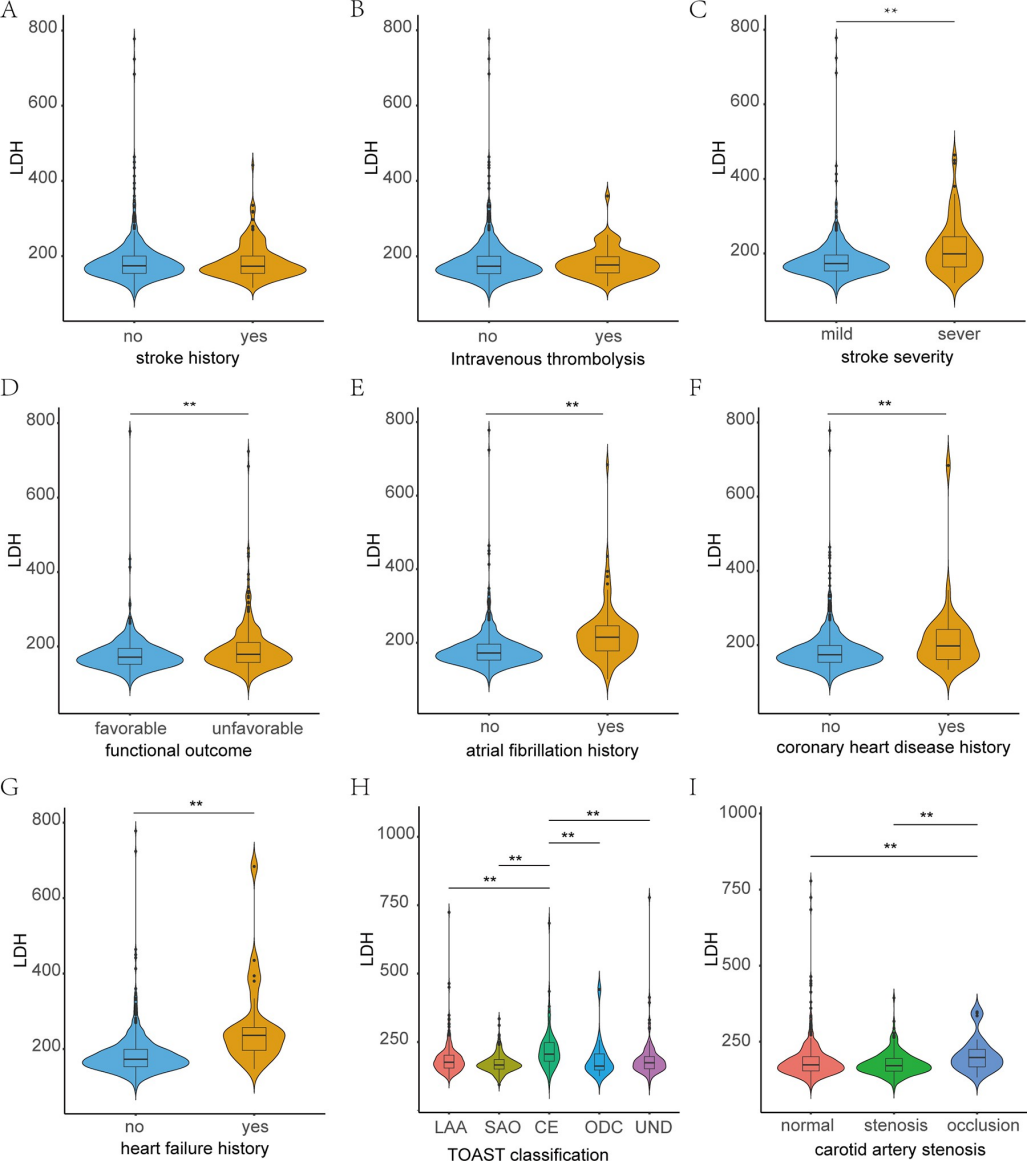
Comparisons of admission LDH levels according to stroke history (A), intravenous thrombolysis (B), stroke severity (C), functional outcome (D), atrial fibrillation (E), coronaty heart disease history (F), heart failure history (G), TOSAT classification (H) and carotid artery stenosis(I) in AIS patients. LAA, Large-artery atherosclerosis; CE, Cardioembolism; SAO, Small-vessel occlusion; ODC, Other determined etiology; UND, Undetermined etiology. *: P<0.05; **: P<0.01.

As shown in Fig 1, a total of 732 patients with AIS were enrolled in our study from July 2019 to December 2019. The median age of all participants was 68 years (IQR, 61–76), and 421(57.5%) cases were men. 179(24.5%) patients had a history of smoking, and 127(17.3%) patients had a history of drinking. Medical history included history of stroke (n = 189, 25.8%), hypertension (n = 541, 73.9%), diabetes mellitus (n = 232, 31.7%), AF (n = 89, 12.2%), HF (n = 25, 3.4%) and CAD (n = 33, 4.5%). 198(27.0%) patients had Carotid artery stenosis and 28(3.8%) patients had carotid artery occlusion. Median NIHSS scores on admission and discharge were 3(IQR, 1–7), 2(IQR, 0–4), median mRS scores on admission and discharge were 3(IQR, 1–4), 2(IQR, 1–3). Median SPI-Ⅱscores was 5(IQR, 3–7). Baseline characteristics of patients were shown in S1 Table. In addition, the LDH levels of AIS patients with mild stroke were significantly lower than that of severe stroke. Similarly, the LDH levels of patients with favorable functional outcomes were significantly lower than those with unfavorable functional outcomes. LDH levels in patients with a history of atrial fibrillation, coronary heart disease, and heart failure were significantly higher than those without a history. LDH in patients with TOAST classification of cardioembolism was significantly higher than that in other groups. LDH in patients with carotid artery occlusion was significantly higher than that in patients with stenosis and normal (Fig 2).

### Association between LDH and poor prognosis in AIS patients

**Fig 3:**
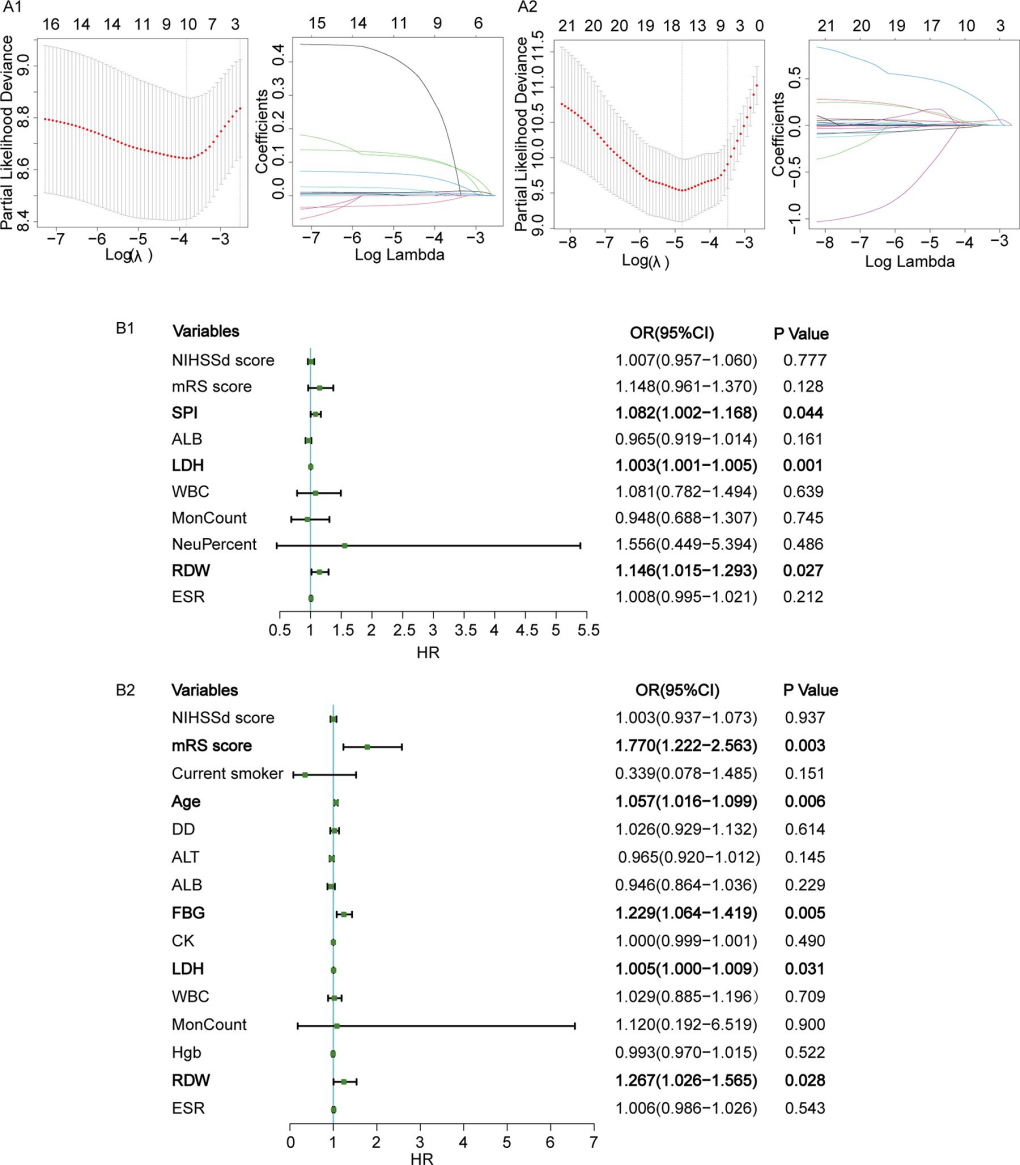
The LASSO Cox proportional hazard regression and forest map of Cox proportional hazard regression for prognosis and all-cause death in patients with AIS. A1. LASSO Cox proportional hazard regression for prognosis in patients with AIS; A2. LASSO Cox proportional hazard regression for all-cause death in patients with AIS; B1. Forest map of Cox proportional hazard regression for prognosis in patients with AIS; B2. Forest map of Cox proportional hazard regression for all-cause death in patients with AIS.

Of the 732 patients, 608 (83.1%) had a favorable prognosis and 124 (16.9%) had a poor prognosis. In univariate analysis, age, stroke history, AF, CHD, admission NIHSS score, discharge NIHSS score, admission mRS score, discharge mRS score, SPI-Ⅱscore, ALB, CK, LDH, DD, Fib, WBC, Neutrophil, Monocyte, Hgb, RDW, and ESR were significantly associated with poor prognosis (p < 0.05). The poor prognosis group had statistically elevated admission LDH level (median, 186; IQR, 159–228) compared to favorable prognosis group(median, 173; IQR, 153–198; P < 0.001) (S1 Table). Lasso Cox regression and multivariate COX regression analyses showed that admission LDH levels were significantly correlated with poor prognosis in patients with AIS (OR, 1.003; 95% CI, 1.001–1.005; P = 0.001) (Fig 3A1 and 3B1).

**Fig 4:**
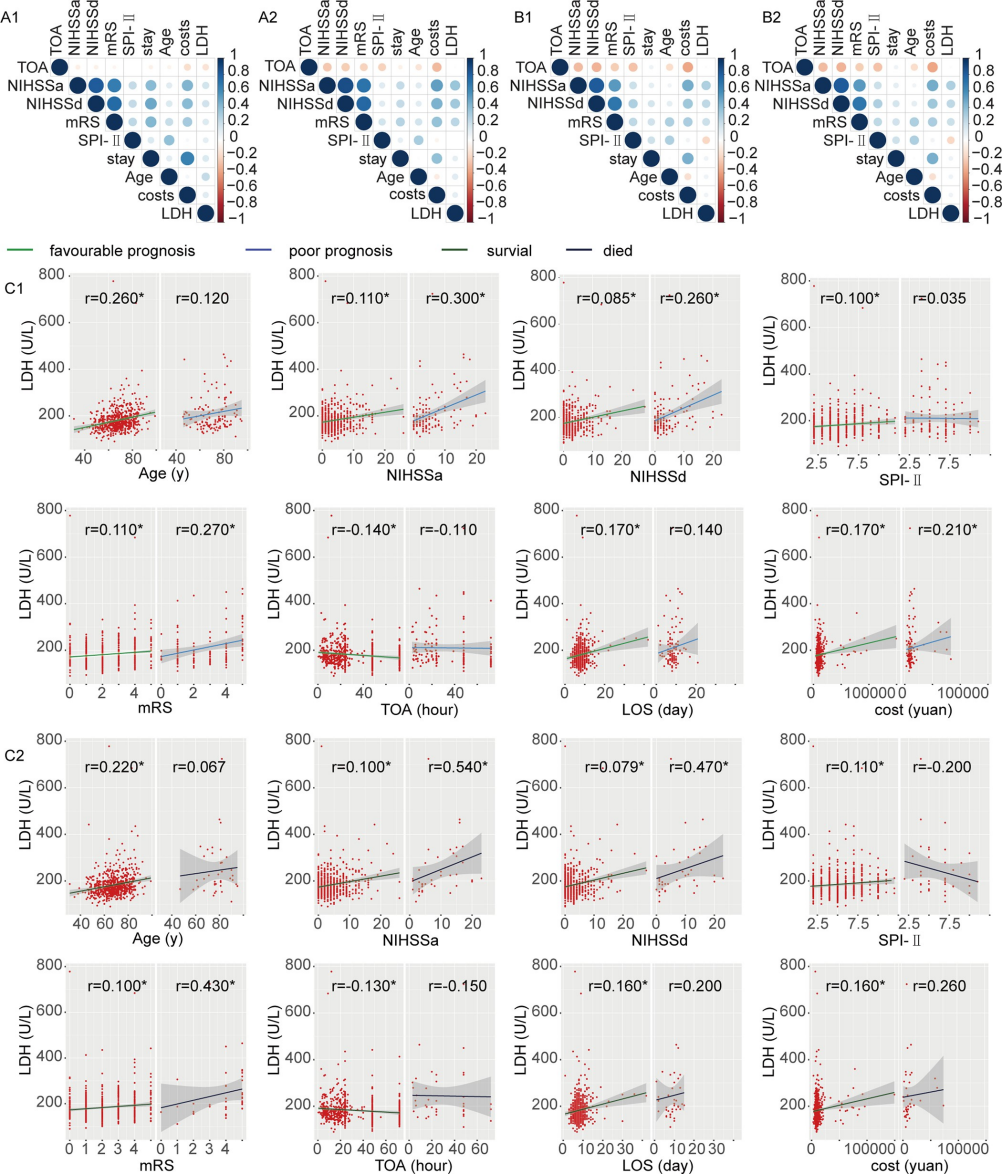
Correlation of LDH with clinical characteristics in AIS patients. A1. Correlation of LDH with clinical characteristics in AIS patients with favourable prognosis; A2. Correlation of LDH with clinical characteristics in AIS patients with poor prognosis; B1. Correlation of LDH with clinical characteristics in survival AIS patients; B2. Correlation of LDH with clinical characteristics in died AIS patients; C1. Comparison of the correlation of LDH and clinical characteristics between AIS patients with favourable prognosis and poor prognosis; C2. Comparison of the correlation of LDH and clinical characteristics between survival AIS patients and died AIS patients. TOA, time from onset to admission; NIHSSa, NIHSS score on admission; NIHSSd, NIHSS score on discharge; LOS, length of stay; cost, cost of hospitalization.

As shown in Fig 4A1, 4A2 and 4C1, admission LDH level was positively correlated with age, admission NIHSS score, discharge NIHSS score, mRS score, SPI-Ⅱscore, time from onset to admission (TOA), length of stay (LOS) and cost in the favorable prognosis group, while in the poor prognosis group, admission LDH level was positively correlated with admission NIHSS score, discharge NIHSS score, mRS score and cost, but not significantly correlated with age, SPI-Ⅱscore, TOA, and LOS.

### Association between LDH and all-cause death in AIS patients

Finally, 18 (2.46%), 27 (3.69%), 36 (4.92%) and 39 (5.33%) patients died at 3-month, 6-month, 12-month and 18-month. In univariate analysis, age, current smoker, AF, HF, CHD, hospitalization costs, admission NIHSS score, discharge NIHSS score, admission mRS score, discharge mRS score, SPI-Ⅱscore, ALT, ALB, FBG, CK, LDH, hs-CRP, DD, Fib, WBC, Neutrophil, Monocyte, RBC, Hgb, RDW, and ESR were significantly associated with all-cause death (p < 0.05). The death group had statistically elevated admission LDH level (median, 204; IQR, 178–302) compared to the survival group (median, 173; IQR, 153–199; P < 0.001) (S1 Table). Lasso Cox regression and multivariate COX regression analyses showed that admission LDH levels were significantly correlated with all-cause death in patients with AIS (OR, 1.005; 95% CI, 1.000–1.009; P = 0.031) (Fig 3A2 and 3B2).

As shown in Fig 4B1, 4B2 and 4C2, admission LDH level was positively correlated with age, admission NIHSS score, discharge NIHSS score, mRS score, SPI-Ⅱscore, time from onset to admission (TOA), length of stay (LOS) and cost in the survival group, while in the death group, admission LDH level was positively correlated with admission NIHSS score, discharge NIHSS score and mRS score, but not significantly correlated with age, SPI-Ⅱscore, TOA, LOS and cost.

### Predictive values of LDH for the poor prognosis of AIS

**Fig 5:**
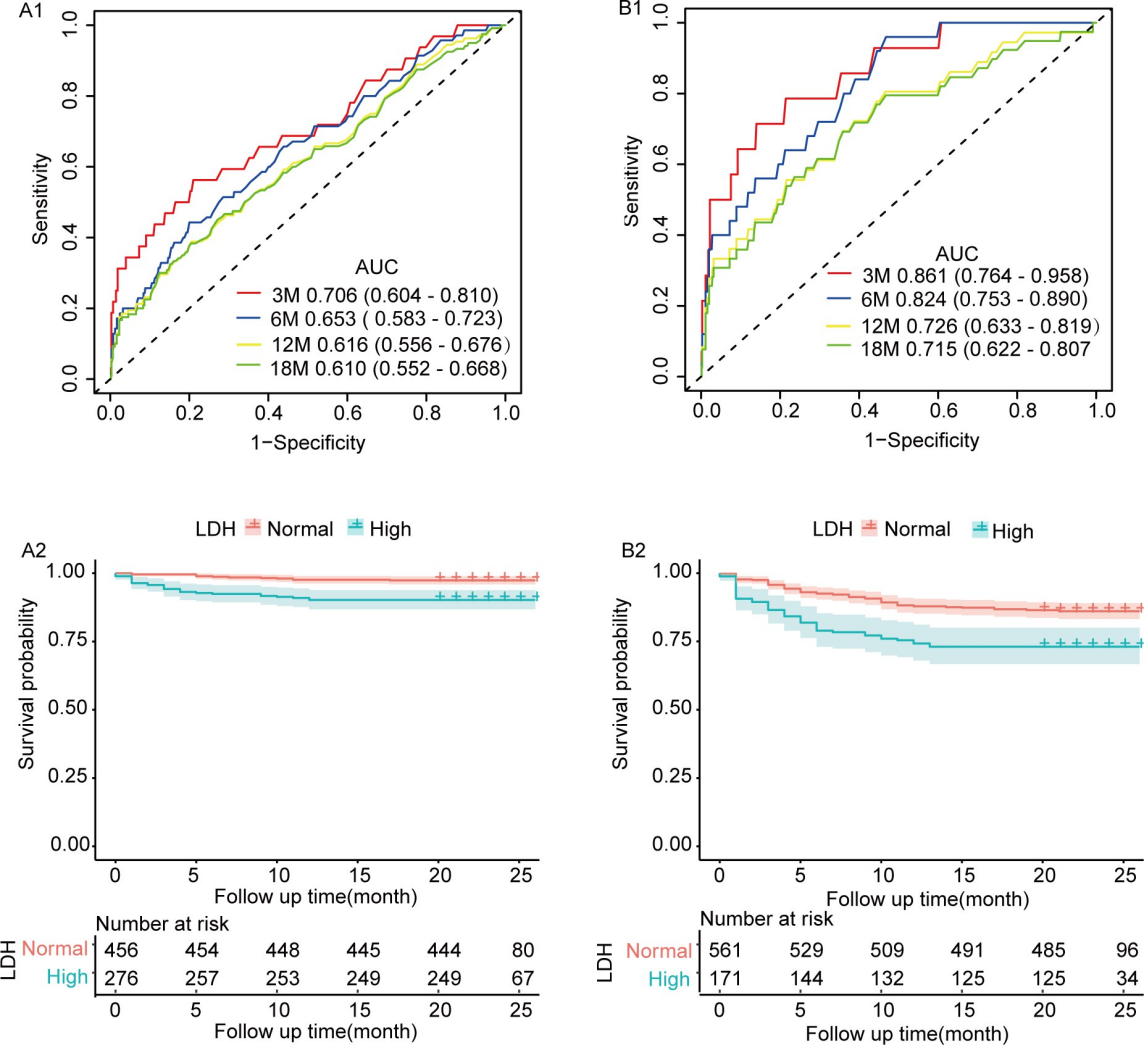
ROC curves and KM curves of LDH for prognosis and all-cause death in patients with AIS. A. ROC curves and KM curves of LDH for prognosis in patients with AIS; B. ROC curves and KM curves of LDH for all-cause death in patients with AIS.

The td-ROC curve showed that the AUC values of admission LDH level for predicting prognosis of AIS patients in 3-month, 6-month, 12-month and 18-month were 0.706 (95% CI, 0.604–0.810), 0.653 (95% CI, 0.583–0.723), 0.616 (95% CI, 0.556–60676) and 0.610 (95% CI, 0.552–0.680), respectively, and the optimal cut-off value of LDH was 202U/L (Fig 5A1). The Kaplan-Meier curve showed that higher admission LDH level was positively correlated with the poor prognosis. The log-rank test revealed a poor prognosis in the high admission LDH level group (P < 0.001) (Fig 5A2).

**Fig 6:**
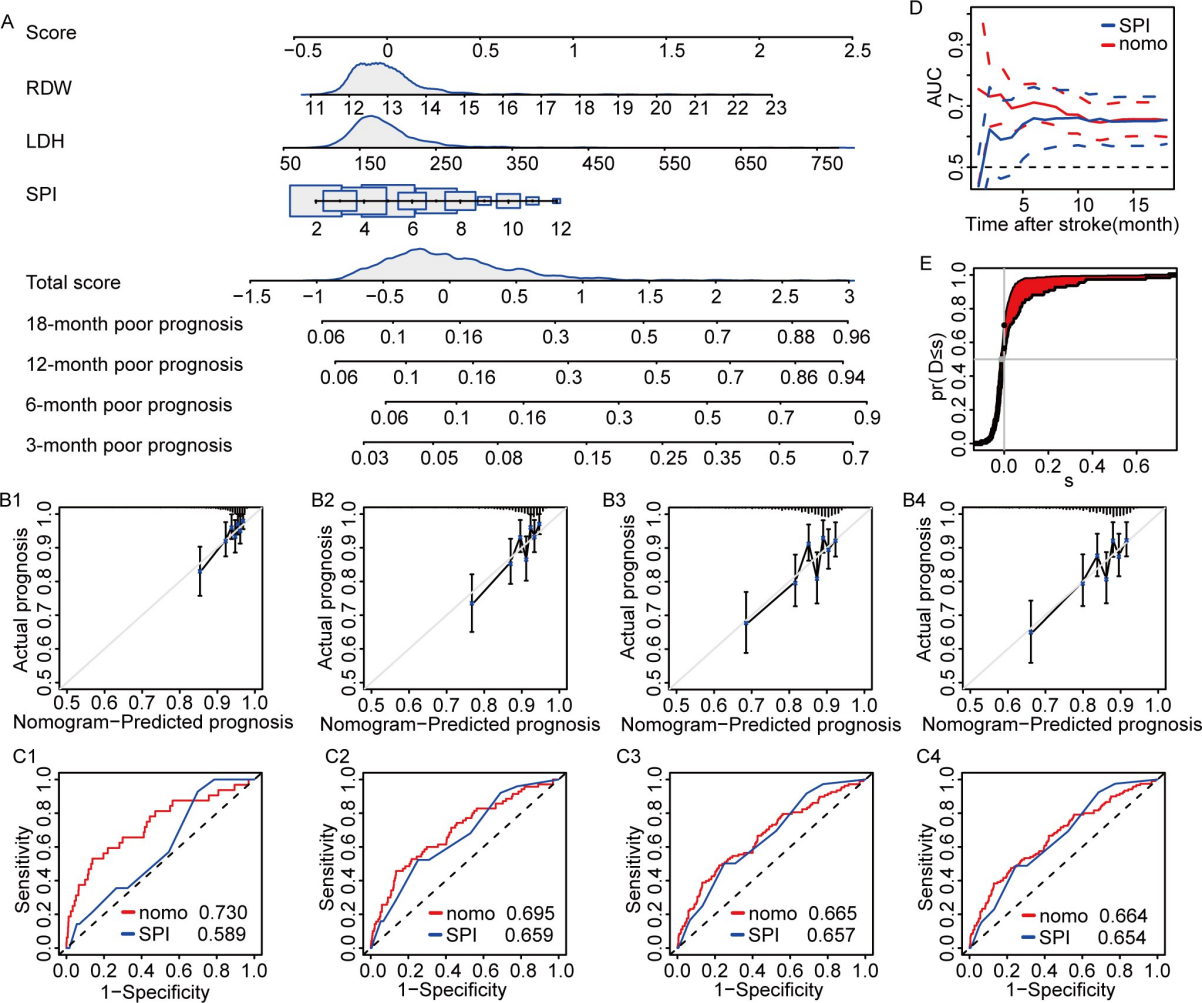
The models for predicting the prognosis in patients with AIS. A. Nomogram for the prognosis in patients with AIS; B. The calibration curves based on nomogram prediction and actual observation at 3-month(B1), 6-month(B2), 12-month(B3), 18-month(B4); C. Time-dependent ROC curves to evaluate the accuracy of the nomogram at 3-month(C1), 6-month(C2), 12-month(C3), 18-month(C4); D. Time-dependent ROC curves to evaluate the accuracy of the nomogram. E. NRI and IDI to evaluate the accuracy between the nomogram and the SPI score.

The nomogram model showed the admission LDH level, admission RDW level, and SPI-Ⅱ score was better in predicting the poor prognosis of AIS. It showed that among AIS patients, the higher the admission LDH level, the worse the prognosis of AIS patients (Fig 6).

### Predictive values of LDH for the all-cause death of AIS

The td-ROC curve showed that the AUC values of admission LDH level for predicting all-cause death of AIS patients in 3-month, 6-month, 12-month, 18-month were 0.861 (95% CI, 0.764–0.958), 0.824 (95% CI, 0.753–0.890), 0.726 (95% CI, 0.633–0.819) and 0.715 (95% CI, 0.622–0.807), respectively, and the optimal cut-off value of LDH was 185U/L. (Fig 5B1). The Kaplan-Meier curve showed that higher admission LDH level was positively correlated with the higher mortality risk. The log-rank test revealed a higher mortality risk in the high admission LDH level group (P < 0.001) (Fig 5B2).

**Fig 7:**
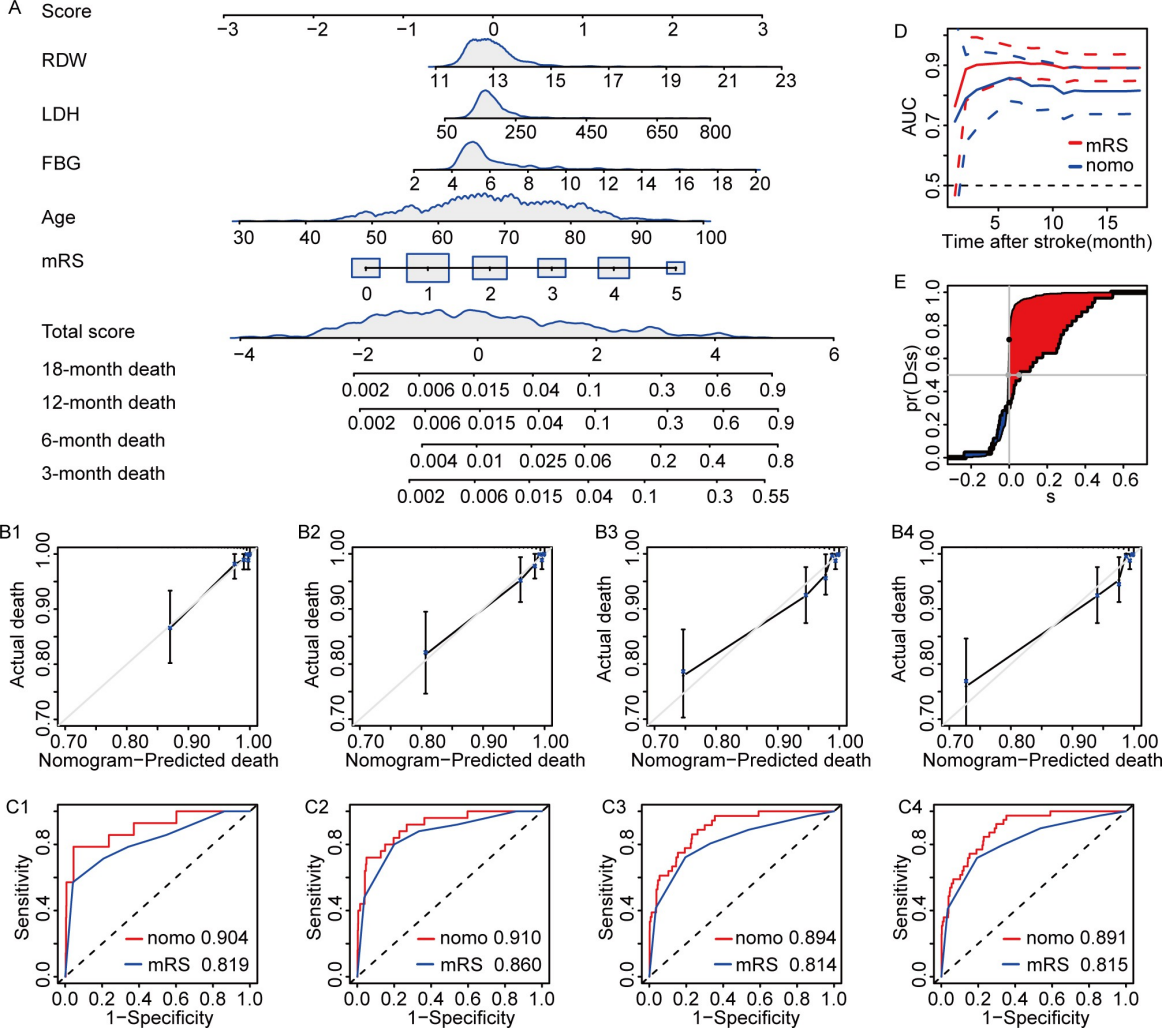
The models for predicting death probability in AIS patients. A. Nomogram for predicting death probability in AIS patients; B. The calibration curves based on nomogram prediction and actual observation at 3-month(B1), 6-month(B2), 12-month(B3), 18-month(B4); C. Time-dependent ROC curves to evaluate the accuracy of the nomogram at 3-month(C1), 6-month(C2), 12-month(C3), 18-month(C4); D. Time-dependent ROC curves to evaluate the accuracy of the nomogram. E. NRI and IDI to evaluate the accuracy between the nomogram and the mRS score.

The nomogram model showed the admission LDH level, admission RDW level, admission FBG level, age, and mRS score were better in predicting the all-cause death of AIS. It showed that among AIS patients, the higher the admission LDH level, the higher the mortality risk of AIS patients (Fig 7).

## Discussion

In this study, we found that patients who relapsed or died 18 months after AIS had higher admission LDH levels, which were significantly correlated with stroke severity and poor prognosis. After further adjustment for important potential confounding factors by multivariate analysis, these associations still exist. Therefore, LDH may be a predictor of the risk of poor prognosis and all-cause death of AIS.

The mortality rates at 3-month, 6-month, 12-month and 18-month were 2.46%, 3.69%, 4.92% and 5.33%, respectively. One study from China National Stroke Registry (CNSR) recording all stroke patients occurred between September 2007 and August 2008 and reported 12-month mortality rate was 13.4% in AIS patients [14], which was higher than our study. Another study from Bigdata Observatory Plat Form for Stroke of China, recording 27316 first-ever Ischemic Stroke patients, reported the 12-month mortality rate was 6.0%, slightly higher than our study [15]. In addition, a study from CNSR-III recording 9128 AIS patients and 668 transient ischemic attack between 2015 and March 2018 in China, reported 329 patients died at 12-month, indicating that the 12-month mortality rate of AIS patients was ≤3.6%, slightly lower than this study [16]. The mortality rate of AIS has been decreasing in recent years. The China Stroke Prevention Project Committee (CSPPC) Stroke program has significantly improved stroke care, which is important in reducing Stroke mortality [17].

LDH is a cytoplasmic enzyme existing in all tissues of the body. It catalyzes the reversible reaction between pyruvate and lactic acid and plays a key role in the process of anaerobic respiration. Serum LDH detected clinically consists of five isozymes. LDH-1 and LDH-2 mainly exist in erythrocytes and myocardium, LDH-3 is mainly expressed in lymphoid tissues, brain, and other tissues, LDH-4 and LDH-5 are mainly expressed in liver, skeletal muscle, and tumor tissues. In some special cases, the LDH isozyme activity spectrum can be transformed [18]. It is reported that many tumor cells have the Warburg effect of obtaining energy by anaerobic fermentation regardless of whether oxygen is sufficient or not. Therefore, LDH can be used as a biomarker for the prognosis of a variety of tumors [19]. It has been reported that the progression-free survival and overall survival of large B-cell lymphoma with elevated LDH levels are short [20]. Li et al. Noted that baseline LDH ≥ 252 μ/ L the recurrence-free survival of patients with locally advanced cervical cancer is poor, indicating that high-level LDH plays a certain role in suggesting the recurrence of cervical cancer [21]. A multicenter study found that high LDH levels could be a sign of a tumor’s recruitment of suppressive and inflammatory cells worrying diagnosis of different types of cancer [22]. Because LDH plays a role in human glucose metabolism, LDH is also closely related to the prognosis of many diseases. Xiang et al. Showed that the LDH level within 24 hours after admission can be used as an indicator to evaluate the severity and prognosis of acute pancreatitis [23]. It has also been reported that LDH levels > 280u / L are associated with increased all-cause mortality and cardiovascular mortality in hemodialysis patients [24].

The above results show that in clinical practice, LDH, as a conventional biomarker that can be easily measured in most clinical laboratories, is convenient and rapid in clinical detection, and is often used to evaluate the prognosis of the disease. However, there are few reports on the relationship between LDH and stroke prognosis. Muiz et al. Pointed out that high-level LDH is a predictor of mortality in patients with intracerebral hemorrhage [25], and Wang et al pointed out that high-level LDH is independently associated with the risk of all-cause death and poor functional prognosis in patients with AIS or TIA [16]. After analyzing the clinical and laboratory data of AIS patients, this study found that AIS patients with high LDH levels have an increased risk of AIS recurrence and all-cause death, which indicates that LDH can predict the recurrence and all-cause death of AIS patients.

At present, the exact mechanism between LDH and AIS recurrence and death is not clear, which may be related to the above reasons. Firstly, the occurrence of AIS is due to the necrosis and softening of brain tissue caused by the disorder of brain blood supply, and then the dysfunction of corresponding functional areas. The increase of LDH is common in tissue injury, necrosis, or hypoxia, which means that the increase of LDH may be related to the recurrence of AIS [26]. Secondly, LDH is considered an important inflammatory biomarker [27], and a large number of studies have proved that inflammation plays an important role in the occurrence, development, and prognosis of cerebral infarction [28,29].

When analyzing the correlation between admission LDH level and clinical data of AIS patients, we found that admission LDH level was positively correlated with NIHSS score and mRS score. This shows that the admission LDH level has a certain suggestive effect on the initial severity and unfavorable functional outcome of AIS patients. In addition, this study also found that the correlation between LDH level and poor prognosis group was better than that of favorable prognosis group. Similarly, the correlation with the death group was better than that of the survival group. LDH is widely distributed in all cells and tissues. When the tissue is damaged, the cell membrane structure, function, and cell membrane permeability change, and a large amount of LDH is released into the blood, which can lead to the increase of serum LDH level [30]. The clear mechanism needs to be further investigated.

We found that although the admission LDH level can be used alone to predict the risk of poor prognosis and all-cause death in AIS patients, the diagnostic efficiency still needs to be further improved. Therefore, we further constructed the nomogram prediction model by using the variables determined by multiple Cox regression analysis. The characteristic of a nomogram is that it can integrate multiple prediction indicators and predict the occurrence probability of an outcome event individually and accurately. The nomogram model established in this study has a diagnostic accuracy of 0.730, 0.695, 0.665, and 0.664 for poor prognosis of AIS patients at 3, 6, 12, and 18 months. The diagnostic accuracy of all-cause death of AIS patients at 3, 6, 12, and 18 months was 0.901, 0.909, 0.894, and 0.892.

There are still some shortcomings in this study. First, we only explored the relationship between LDH levels and AIS at admission, and the lack of dynamic observation study. Second, all the cases included in this study were from the same hospital, the sample size was relatively small, and there might be a particular bias. Finally, some potential indicators, such as the size of the stroke area, and other biomarkers of inflammation(i.e.,IL-6, TNF-α) were not included in the study.

In conclusion, a high level of LDH is an independent predictor of the risk of poor prognosis and all-cause death of AIS and has a certain suggestive effect on the initial severity and unfavorable functional outcome of AIS patients. In the future, we should carry out a large sample and multi-center prospective study to further explore the prognostic value of LDH in AIS, to provide a basis for the diagnosis and treatment of AIS.

## Conclusions

In conclusion, High admission LDH level was an independent predictor of the poor prognosis or all-cause death of AIS and was suggestive of the initial severity and unfavorable functional outcome of AIS patients. LDH may be a good predictor due to the applicability and simplification for routine use based on common clinical practice. Patients with high LDH levels should be closely followed up, and timely intervened to prevent AIS recurrence or death.

## Supporting information

S1 Table Clinical characteristics of AIS patients. TOA, Time from onset to admission; NIHSS, The National Institutes of Health Stroke Scale; mRS, Modified Rankin Scale; SPI-Ⅱ, Stroke Prognostic Instrument Ⅱ; ALT, alanine aminotransferase; AST, aspartate aminotransferase; TP, total protein; ALB, albumin; FBG, fasting blood glucose; TC, total cholesterol; TG, triglyceride; LDL, low-density lipoprotein; HDL, high-density lipoprotein; CK, creatine kinase; LDH, lactate dehydrogenase; hs-CRP, high-sensitivity C-reactive protein; DD, D dimer; Fib, fibrinogen; INR, international normalized ratio; Wbc, white blood cell; RBC, red blood cell; Hgb, hemoglobin; RDW, red blood cell distribution; PLT, platelet; ESR, erythrocyte sedimentation.
(PDF)

S2 Table (XLS)
