# Supplementary material for: Elevated lactate dehydrogenase predicts poor prognosis of acute ischemic stroke
Source: PLoS One. 2022 Oct 7;17(10):e0275651. doi: 10.1371/journal.pone.0275651 (PMC9544033; doi:10.1371/journal.pone.0275651)
Supplement: S1 Table — TOA, Time from onset to admission; NIHSS, The National Institutes of Health Stroke Scale; mRS, Modified Rankin Scale; SPI-Ⅱ, Stroke Prognostic Instrument Ⅱ; ALT, alanine aminotransferase; AST, aspartate aminotransferase; TP, total protein; ALB, albumin; FBG, fasting blood glucose; TC, total cholesterol; TG, triglyceride; LDL, low-density lipoprotein; HDL, high-density lipoprotein; CK, creatine kinase; LDH, lactate dehydrogenase; hs-CRP, high-sensitivity C-reactive protein; DD, D dimer; Fib, fibrinogen; INR, international normalized ratio; Wbc, white blood cell; RBC, red blood cell; Hgb, hemoglobin; RDW, red blood cell distribution; PLT, platelet; ESR, erythrocyte sedimentation. (PDF) [file pone.0275651.s001.pdf]

S1 Table Clinical characteristics of AIS patients.

Variables	Overall n = 732	Prognosis: Favourable prognosis n = 608	Prognosis: Poor prognosis n =124	P Value	Survival state: Survivors n = 693	Survival state: Death n = 39	P Value
Age, y	68.0(61-76)	68(61-75)	72(63-81)	0.002	68(61-75)	79(71-82)	<0.001
Men, n(%)	421(57.5)	344(56.6)	77(62.1)	0.285	404(58.3)	2(5.1)	0.084
Current smoker, n(%)	179(24.5)	153(25.2)	26(21.0)	0.308	177(25.5)	5(12.8)	0.013
Current drinker, n(%)	127(17.3)	106(17.4)	21(16.9)	0.894	122(17.6)	17(43.6)	0.454
Medical history, n(%)							
Stroke history	189(25.8)	140(23.0)	49(39.5)	<0.001	176(25.4)	13(33.3)	0.237
Hypertension	541(73.9)	453(74.5)	88(71.0)	0.432	514(74.2)	27(69.2)	0.495
Diabetes mellitus	232(31.7)	188(30.9)	44(35.5)	0.310	220(31.7)	12(30.8)	0.936
Atrial Fibrillation	89(12.2)	63(10.4)	26(21.0)	0.001	75(10.8)	14(35.9)	<0.001
Heart failure	25(3.4)	19(3.1)	6(4.8)	0.295	21(3.0)	4(10.3)	0.019
Coronary heart disease	33(4.5)	20(3.3)	13(10.5)	<0.001	28(4.0)	5(12.8)	0.008
Carotid artery stenosis, n(%)				0.210			0.860
Stenosis	198(27.0)	166(27.3)	32(25.8)		195(28.1)	3(7.7)	
Occlusion	28(3.8)	19(3.1)	9(7.3)		23(3.3)	5(12.8)	
TOAST, n(%)				0.994			0.848
Large-artery atherosclerosis	261(35.7)	207(34.0)	54(43.5)		245(35.4)	16(41.0)	
Cardioembolism	75(10.2)	52(8.6)	23(18.5)		62(8.9)	13(33.3)	
Small-vessel occlusion	245(33.5)	224(36.8)	21(16.9)		241(34.8)	4(10.3)	
Other determined etiology	17(2.3)	12(2.0)	5(4.0)		17(2.5)	0(0.0)	
Undetermined etiology	134(18.3)	113(18.6)	21(16.9)		128(18.5)	6(15.4)	

Medication in hospital, n (%)							
Intravenous thrombolysis	69(9.40)	60(9.9)	9(7.3)	0.379	64(9.2)	5(12.8)	0.488
TOA, h	24.0(15.0-48.0)	24.0(15.0-48.0)	24.0(15.0-48.0)	0.992	24.0(15.0-48.0)	24.0(14.5-48.0)	0.582
length of stay, d	8(7-9)	8(6-9)	8(7-10)	0.572	8(7-9)	9(7-11)	0.204
hospitalization costs, yuan	9092.6 (7703.8-11033.6)	8905.2 (7682.1-10861.5)	9417.0 (7803.6-12048.1)	0.099	8956.3 (7684.1-10832.0)	10970.1 (8117.0-15797.5)	0.019
NIHSS score on admission	3(1-7)	3(1-5)	4(2-8)	<0.001	3(1-5)	7(4-15)	<0.001
NIHSS score on discharge	2(0-4)	2(0-3)	3(1-6)	<0.001	2(0-3)	8(2-13)	<0.001
mRS score on admission	3(1-4)	2(1-4)	4(2-4)	<0.001	2(1-4)	4(3-5)	<0.001
mRS score on discharge	2(1-3)	2(1-3)	3(1-4)	<0.001	2(1-3)	4(3-5)	<0.001
SPI-Ⅱscore	5(3-7)	5(3-6)	5(4-7)	<0.001	5(3-7)	5(4-7)	0.001
ALT, U/L	16(11-22)	16(11-21)	15(11-23)	0.790	16(11-22)	13(9-19)	0.047
AST, U/L	20(17-25)	20(17-25)	23(18-29)	0.657	20(17-25)	24(18-34)	0.498
TP, g/L	65.0(61.3-68.5)	65.0(61.3-68.3)	64.6(61.0-68.9)	0.838	65.0(61.3-68.5)	65.0(59.3-69.5)	0.879
ALB, g/L	38.5(36.3-40.4)	38.5(36.5-40.5)	38.0(35.5-39.7)	0.001	38.5(36.5-40.4)	36.8(33.3-39.2)	<0.001
FBG, mmol/L	5.40(4.87-6.62)	5.39(4.86-6.56)	5.50(4.91-6.76)	0.554	5.39(4.87-6.51)	6.42(4.89-8.14)	0.011
TC, mmol/L	4.06(3.40-4.79)	4.08(3.41-4.83)	3.91(3.24-4.67)	0.095	4.06(3.41-4.79)	3.88(2.98-4.92)	0.125
TG, mmol/L	1.28(0.92-1.88)	1.29(0.93-1.90)	1.19(0.86-1.73)	0.128	1.29(0.93-1.89)	1.11(0.63-1.56)	0.109
LDL, mmol/L	2.19(1.70-2.76)	2.21(1.71-2.77)	2.12(1.52-2.73)	0.139	2.20(1.72-2.77)	1.97(1.33-2.61)	0.065
HDL, mmol/L	1.19(1.02-1.35)	1.19(1.02-1.34)	1.19(1.02-1.37)	0.984	1.18(1.02-1.34)	1.33(1.00-1.49)	0.216
CK, U/L	75(55-110)	75(54-110)	76(56-127)	0.006	75(55-109)	69(56-163)	<0.001
LDH, U/L	174(154-200)	173(153-198)	186(159-228)	<0.001	173(153-199)	204(178-302)	<0.001
hs-CRP, mg/L	2.20(1.13-5.60)	2.20(1.10-4.98)	4.30(1.19-10.85)	0.056	2.20(1.10-5.15)	10.70(2.40-22.70	0.003
DD, mg/L	0.47(0.32-0.91)	0.46(0.32-0.87)	0.55(0.36-1.06)	0.004	0.46(0.32-0.87)	0.93(0.53-1.66)	<0.001
Fib, g/L	3.16(2.78-3.76)	3.10(2.74-3.66)	3.42(2.96-4.02)	0.001	3.14(2.77-3.70)	4.00(3.08-4.44)	0.002
INR	1.02(0.98-1.08)	1.02(0.98-1.07)	1.04(0.99-1.10)	0.205	1.02(0.98-1.07)	1.06(1.02-1.12)	0.933

Wbc, 10*^{9}/L	6.22(5.15-7.69)	6.15(5.09-7.58)	7.05(5.29-8.74)	<0.001	6.18(5.13-7.58)	7.70(5.82-9.64)	<0.001
Neutrophil, 10*^{9}/L	4.08(3.07-5.22)	4.02(3.067-5.09)	4.65(3.31-6.07)	<0.001	4.02(3.067-5.09)	5.91(3.73-7.63)	<0.001
Lymphocyte, 10*^{9}/L	1.49(1.16-1.85)	1.48(1.18-1.87)	1.50(1.12-1.81)	0.187	1.49(1.17-1.85)	1.45(0.91-1.87)	0.078
Monocyte, 10*^{9}/L	0.40(0.33-0.52)	0.40(0.32-0.50)	0.45(0.35-0.57)	0.001	0.40(0.33-0.51)	0.56(0.35-0.63)	<0.001
RBC, 10*^{12}/L	4.32(3.97-4.66)	4.33(3.98-4.65)	4.26(3.87-4.69)	0.253	4.33(3.99-4.66)	4.02(3.60-4.44)	0.004
Hgb, g/L	131(120-141)	132(120-142)	126(117-139)	0.020	131(121-141)	117(110-128)	<0.001
RDW, %	12.8(12.3-13.3)	12.8(12.3-13.3)	13.0(12.4-13.7)	<0.001	12.8(12.3-13.3)	13.2(12.8-14.2)	<0.001
PLT, 10*^{9}/L	217(178-258)	218(179-256)	216(178-262)	0.263	218(180-258)	208(175-252)	0.770
ESR, mm/H	9(4-17)	9(4-16)	14(6-26)	<0.001	9(4-16)	24(12-36)	<0.001

TOA, Time from onset to admission; NIHSS, The National Institutes of Health Stroke Scale; mRS, Modified Rankin Scale; SPI-Ⅱ, Stroke Prognostic Instrument Ⅱ; ALT, alanine aminotransferase; AST, aspartate aminotransferase; TP, total protein; ALB, albumin; FBG, fasting blood glucose; TC, total cholesterol; TG, triglyceride; LDL, low-density lipoprotein; HDL, high-density lipoprotein; CK, creatine kinase; LDH, lactate dehydrogenase; hs-CRP, high-sensitivity C-reactive protein; DD, D dimer; Fib, fibrinogen; INR, international normalized ratio; Wbc, white blood cell; RBC, red blood cell; Hgb, hemoglobin; RDW, red blood cell distribution; PLT, platelet; ESR, erythrocyte sedimentation.
